# Increasing Incidence of Pediatric Type 1 Diabetes Mellitus in Southeastern Wisconsin: Relationship with Body Weight at Diagnosis

**DOI:** 10.1371/journal.pone.0006873

**Published:** 2009-09-03

**Authors:** Jennifer Evertsen, Ramin Alemzadeh, Xujing Wang

**Affiliations:** 1 Departments of Epidemiology & Biostatistics Medical College of Wisconsin, Milwaukee, Wisconsin, United States of America; 2 University of Wisconsin-Madison, Center for Urban Population Health, Milwaukee, Wisconsin, United States of America; 3 Department of Pediatrics, Medical College of Wisconsin, Milwaukee, Wisconsin, United States of America; Mayo Clinic College of Medicine, United States of America

## Abstract

**Introduction:**

Several studies have confirmed the increasing rate of type 1 diabetes mellitus (T1DM) in children and the link with increasing BMI at diagnosis termed the ‘accelerator hypothesis’. Our objective was to assess whether changing incidence of type 1 diabetes in a group of children and adolescent from the Midwest United States was associated with changes in BMI.

**Methods:**

Data from 1618 (52.1% M/47.9% F) newly-diagnosed children and adolescents (<19 years) with T1DM, admitted to Children's Hospital of Wisconsin (CHW) between January 1995 and December 2004, was analyzed in relationship to body mass index (BMI) standard deviation score (SDS).

**Results:**

An overall, 10-year cumulative incidence of 27.92 per 100,000 (19.12 to 41.72/100,000) was observed, with an average yearly cumulative incidence of 2.39%. The increase was largest in the younger age groups, 0–4, 5–9, and 10–14 having an average yearly increase of 2.4, 2.3, and 3.0%, respectively, corresponding to a relative 10-year increase of 25.3, 33.8, and 38.0%, respectively. Age at diagnosis was inversely correlated with BMI SDS (p<0.001) and remained significant for both males and females.

**Conclusions:**

Annual incidence of T1DM increased two-fold at CHW over the 10-year study period. The majority of the increase was observed in the youngest age groups, which also appeared to be the heaviest. This research adds to the growing literature supporting the hypothesis that excess weight gain during childhood may be a risk factor for early manifestation of T1DM.

## Introduction

Type 1A diabetes mellitus (T1DM), an autoimmune disorder, accounts for 10% of diabetes diagnoses, affecting approximately 1.4 million people in the United States (US) and 10–20 million worldwide [1]–[3]. In the United States, 30,000 new cases occur annually and 40% of patients diagnosed are under the age of 20 [2]–[4]. Recently, studies suggest that the incidence of T1DM may be on the rise and increasing incidence in younger children is of the greatest concern.

Incidence rates of pediatric T1DM vary widely throughout the world. Onkamo et al., (1999) reviewed pooled data from 37 studies (from 1960 to 1996), and observed an overall 2.8% to 3.0% per year global increase in incidence of T1DM [5]. However, only one study, by Kostraba et al., (1992) suggested a slightly negative, but not significant, trend in T1DM incidence in children and adolescents [6]. The World Health Organization's DIAMOND study reported incidence rates from over 100 Centers ranging from 0.1/100,000 per year in China and Venezuela to 37.8/100,000 per year in Sardinia and 42.9/100,000 per year in Finland [2]. A large number of studies have been published supporting the rising incidence of T1DM, especially in the younger age groups [7]–[19]. One of the most notable and recent, in the United States, includes a population-based study of incidence rates of T1DM from 10 study locations by The SEARCH for Diabetes in Youth Study. The Search Group found an overall incidence of T1DM in children 0–19 of 24.3 per 100,000 person years with the highest rates observed among the 5–9 and 10–14 age groups with rates of 22.9 and 33.9 per 100,000 respectively [14]. There is still some speculation as to whether there is also an increase in incidence in the older adolescent groups.

While the autoimmune nature of T1DM continues to be under investigation [20], [21], the underlying mechanisms responsible for the rise of T1DM, especially in the younger age groups, remain unknown. However, the “accelerator hypothesis” proposed by Wilkin, is one of the more compelling theories [22]–[24]. This investigator suggested that increasing body weight in younger children acts as an accelerator mechanism for an increased risk of developing T1DM. In fact, an inverse relationship was found between age at diagnosis and body mass index (BMI) at diagnosis and at 12 months after diagnosis, as well as weight at diagnosis and weight change since birth. Essentially, the age at diagnosis becomes younger as children become heavier; suggesting that being overweight accelerates insulin resistance, leading to the development of T1DM in genetically-predisposed individuals. Thereafter a number of papers have been published supporting Wilkin's accelerator hypothesis' [24]–[30]. A study by Libman et al., (2003) in the United States showed an overall significant increase in the prevalence of being overweight in children with T1DM from 12.6% (1979–1989) to 36.8% (1990–1998). However, the older adolescent population (>11 years) was more overweight than the younger children [31]. To date, the role of increasing body weight in very young children as a risk factor for early development of T1DM remains inconclusive.

The increase in incidence of T1DM in younger age groups in relation to increasing BMI has not been confirmed in the literature. Therefore, we set out to determine the changing burden of T1DM in specific age cohorts in relationship to BMI and body weight at diagnosis in Southeastern Wisconsin.

## Methods

### Ethics Statement

This study was approved by the institutional review board (IRB) of Children's Hospital Wisconsin for the retrospective review of patients' clinic charts and, therefore, no informed consent was required. Details that might disclose the identity of the subjects were omitted from data collection.

### Subjects

Data from 1618 children and adolescents with newly diagnosed T1DM, who were evaluated (inpatients and outpatients) at Children's Hospital of Wisconsin (CHW) Diabetes Center (affiliated with Medical College of Wisconsin) between January 1995 and December 2004, were included in the study. Children's Hospital of Wisconsin is the primary source for pediatric diabetes care in the region of Southeastern Wisconsin. No more than 2.8% of all pediatric admissions for any disease are seen at the other 10 area hospitals. Referral rates for new onset patients with diabetes from primary care physicians remained the same throughout the 10-year study period [32]. Therefore, we ascertain that we identified the majority of pediatric patients with new-onset T1DM.

The diagnosis of T1DM was ascertained based on physician diagnosis extracted from chart review. Patients with a physician-diagnosis of Type 2 Diabetes Mellitus or other endocrinology disorder were excluded from the study. We cross-referenced the hospital medical records with clinic medical records to make sure that we had captured all the diabetes patients. The date of admission (date of diagnosis), date of birth, gender, race, diabetic ketoacidosis status at diagnosis, weight, height, BMI, and medical identification number for each patient were extracted from the patients medical record. Growth parameters were assessed at initial evaluation (admission to hospital), including height, weight, and body mass index (BMI, kg/m^2^). BMI was calculated by using weight and height, which were normalized for age and sex by calculating standard deviation scores (SDS) (z-scores) using 2000 Centers for Disease Control and Prevention (CDC) growth charts as a reference standard. Body mass index (BMI) and SDS calculations were determined using the Epi Info nutrition calculator [33].

Subjects were divided into four age cohorts according to age at diagnosis: 0–4, 5–9, 10–14, and 15–19 years and further divided into BMI percentile categories: underweight (≤5^th^), normal (6^th^–85^th^), overweight (86^th^–95^th^), obese (≥96^th^) [34]. The denominator for the analysis was the number of children 0–19 years of age who were located within the 8-county (population: 586,080) study region of SE Wisconsin [35]. This region was chosen by mapping the T1DM patients by zip code. The overall age- and sex-adjusted incidence rates were calculated using the yearly US census data [36].

### Statistical Analysis

Initial descriptive analysis was conducted to summarize data. Data is expressed as mean±SE when appropriate. Rates were calculated using yearly census data. BMI and weight were converted into SDS. SDS is a type of z-score that normalizes age and sex of the research population based on 2000 CDC growth charts. Linear and logistic multivariate regression was used to determine which variables were associated with BMI for age SDS. Variables introduced into the regression analysis included sex, age at diagnosis, year of diagnosis, and presence of ketoacidosis. A p-value of less than or equal to 0.05 was considered significant. Statistical analysis was conducted by using NCSS [37].

## Results

A total of 1618 (52.1% male; 47.9% female) new cases of T1DM were identified among children aged 0–19 during the study period. The ethnic distribution was 80.1% Caucasians (n = 1296), 10.8% African Americans (n = 175), 4.2% Hispanic (n = 68), and 4.9% other ethnicities (Asians/Inuit Indians) (n = 79). The overall cumulative incidence from 1995 to 2004 was 27.92/100,000 (95% CI: 262.8–289.8) (from 19.12 to 41.72/100,000; p = 0.006) for SE Wisconsin. Males have a slightly higher overall 10-year incidence than females. A significant increase in the second half of the study period, between 2000 and 2004, from 23.54 per 100,000 a year to 41.72 per 100,000 per year was found, which corresponds with an increase of 20% for this five-year period (p<0.001) as seen in table 1.

**Table 1:** Yearly incidence (0–19 yrs) of Type 1 DM from 1995 to 2004 in Southeastern Wisconsin.

Year	ALL Cases	ALL Incidence	Males Cases	Males Incidence	Female Cases	Female Incidence
**1995**	110	18.8	57	19.02	53	18.51
**1996**	126	21.5	65	21.69	61	21.3
**1997**	117	20.0	65	21.69	52	18.16
**1998**	120	20.5	55	18.35	65	22.7
**1999**	135	23.0	73	24.35	62	21.65
**2000**	138	23.5	78	26.02	60	20.95
**2001**	181	30.9	86	28.69	95	33.18
**2002**	214	36.5	117	39.03	97	33.88
**2003**	241	41.1	120	40.04	121	42.26
**2004**	236	40.3	127	42.37	109	38.07
**1995–1999**	608	103.7	315	105.1	293	102.3
**2000–2004**	1010	172.3	528	176.2	482	168.3
**1995–2004**	1618	276.0	843	281.3	775	270.6

* Incidence calculated by using yearly U.S census data for 8 county metro area [35].

**Figure 1:**
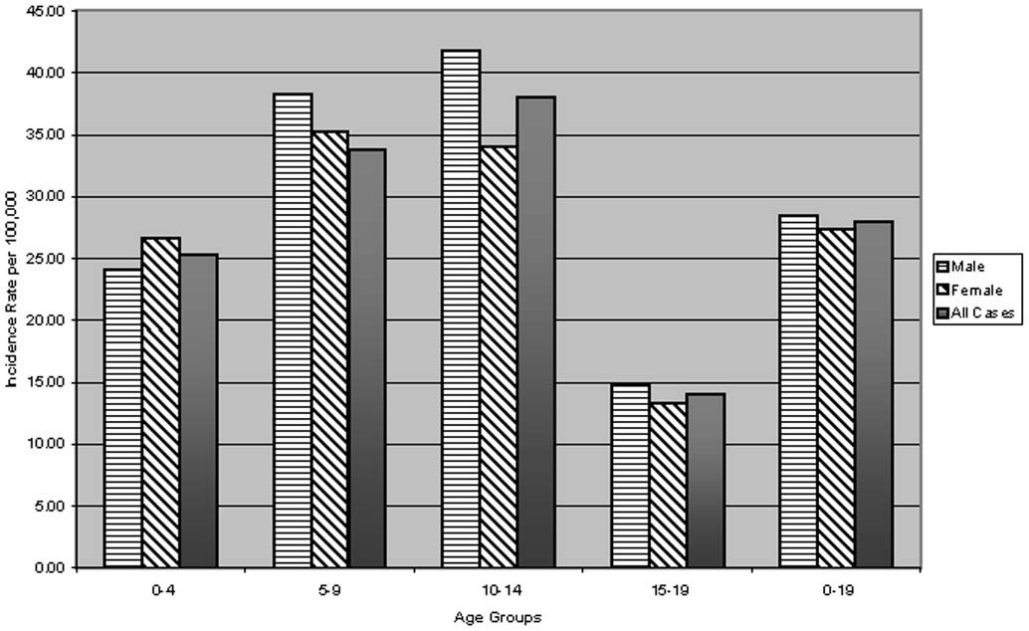
Overall 10-year age (0–19 years) and gender specific incidence. Incidence calculated by using yearly U.S census data for 8 county metro area [35].

The increase in incidence was largest in the younger age groups. The children aged 0–4, 5–9, and 10–14 years, had an increase of 2.4, 2.3, and 3.0% per year, respectively and an overall 10-year relative increase of 25.3, 33.8, and 38.0%, respectively. In the older children (15–19 years), the increase was 1.8% per year with an overall 10-year relative increase of 14.0% as seen in figure 1.

**Figure 2:**
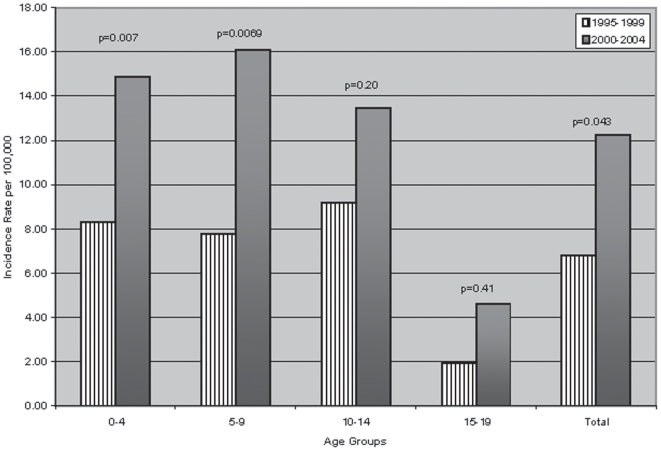
Rates of BMI SDS above 85th (overweight & obese) percentiles for each 5-year period (1995–1999 and 2000–2004) by age. Incidence calculated by using yearly U.S census data for 8 county metro area [35].

A similar increase was seen in both males and females. Females had a slightly higher mean incidence in the younger age groups (0–4 and 5–9 years), and males had a higher mean incidence in the older age groups (10–14 and 15–19 years). Additionally, we calculated the proportion of children and adolescents with BMI SDS above 86^th^ (overweight) and 96^th^ (obese) percentiles from 1999 to 2004. Twenty-eight point four were considered either overweight or obese in this cohort with children ≤9 years, accounting for 60.3 (BMI SDS) and 55.2% (weight SDS) of newly-diagnosed T1DM patients in this category. There also appeared to be a sharp increase in the number of children both overweight and obese during the second five-year period. In fact, the combined rate of overweight (>86^th^ percentile for age) and obese (>96^th^ percentile for age) was significantly increased in the period 2000–2004 for 0–4 year (p = 0.007) and 5–9 year (p = 0.007) age groups, but not for the remainder of the cohort as seen in figure 2.

In the entire cohort, BMI SDS at diagnosis and weight for age were highly correlated (r = 0.61, p<0.001). There were inverse and statistically significant relationships for BMI SDS (p<0.001) at diagnosis in relation to age at diagnosis, based on F-statistic one-way ANOVA. BMI SDS remained significant when separating males and females (males' p = 0.03; females' p = 0.03). A stepwise regression analysis was performed, and BMI SDS was selected (BMI SDS, p = 0.037). In the multiple regression models when using age at diagnosis as the dependent variable, BMI SDS remained significant (BMI r = 0.19, p = 0.04). Age at diagnosis remained significant when using weight SDS as the dependent variable (p<0.001). Although when BMI SDS was used as the dependent variable, age at diagnosis was no longer significant (p = 0.15). penultimate.

## Discussion

Our study results suggest that the overall incidence of T1DM in SE Wisconsin increased two-fold from 1995 to 2004. The largest overall increase in incidence occurred among the youngest age groups of 0–4, 5–9 and 10–14 years, which is consistent with other studies published included the SEARCH group [7]–[19]. Similar to previous studies, females had a slightly higher mean incidence in the younger age groups (0–4 and 5–9 years) than males, whereas males had higher mean incidence than females in the older age groups (10–14 and 15–19 years) [7]–[19]. Indeed, the incidence of T1DM in our cohort in 2004 appeared to be similar to that observed in Finland [9] and the SEARCH Group [14]. Furthermore, the youngest age groups seem to also be the heaviest, in relation to BMI SDS. This relationship seemed to become more prevalent throughout the 10-year study period. Our cohort had a slightly higher number of children and adolescents considered overweight (19.7, BMI SDS; 21.8, weight SDS) and obese (8.53, BMI SDS; 12.67, weight SDS) as compared to the State of Wisconsin's averages of 14 overweight and 10 percent obese [38].

Wilkin (2001) suggested that three accelerators might be responsible: an impending β-cell death, insulin resistance, and a genetic propensity to develop β-cell autoimmunity [22]. Recently, Knerr et al., (2005) also reported that higher BMI was associated with a younger age at diabetes onset in a large cohort of German and Austrian children with T1DM [28]. In our cohort, there was not only an inverse relationship between the age of diagnosis and BMI SDS, but also a two-fold increase in BMI SDS from our second five-year period (2000–2004) in all of the age cohorts as compared with the first five-year period (1995–1999). This increase corresponded with a 20% increase in the proportion of newly-diagnosed children with T1DM during the same period (2000–2004).

There were a number of limitations to this study. First, case ascertainment methods were limited. Cross-reference of all patients diagnosed with diabetes in the hospital medical records was compared with clinic “shadow” records to ascertain that we had captured all the diabetes patients and to verify the T1DM diagnosis. Children's Hospital of Wisconsin (CHW) provides in-patient care to 86% to 90% of pediatric population in the SE Wisconsin region [35]. Consequently, CHW Diabetes Center is believed to capture similar percentages of children and adolescents with T1DM, therefore we believe that our results can be generalizable to other pediatric populations. The referral rates to this hospital for “all treatments” and specifically for “diabetes” remained constant during the 10-year study period. In order to verify that the children in our cohort corresponded with the 8-county metro area we mapped the zip codes recorded in their medical records. Secondly, we were only able to collect one point of height and weight data at diagnosis, which doesn't take into account the possibility of weight loss due to symptoms related to the onset of the diabetes. Therefore, it would have been more meaningful if weight and height data from six months before or after diagnosis were available to evaluate the relationship between age at onset and body weight. Thirdly, we were not able to evaluate β-cell function and number of diabetes antibodies in relationship to age at diagnosis and body weight. Indeed, obesity-induced insulin resistance is believed to up-regulate the β-cells, which become susceptible to an autoimmune attack [39]. These limitations would suggest that our data may be an under-representation of the number of children newly diagnosed with T1DM.

Our study was the first 10-year retrospective cohort in the Midwest, looking at the burden of T1DM and the relationship with BMI. Our findings suggest that the overall incidence of T1DM is increasing in Southeastern Wisconsin, with the largest increase seen in children ≤14 years of age. A likely mechanism for this increase is an increase in weight gain at diagnosis, namely “accelerator hypothesis” [22].

Our research adds to the growing literature that emphasizes the importance of maintaining a healthy weight throughout childhood; which could delay the onset on T1DM into adolescence. Indeed, our data reaffirms that excess weight during early childhood could be an important mechanism in understanding the etiology of T1DM.
